# Predicting cell invasion in breast tumor microenvironment from radiological imaging phenotypes

**DOI:** 10.1186/s12885-021-08122-x

**Published:** 2021-04-07

**Authors:** Dooman Arefan, Ryan M. Hausler, Jules H. Sumkin, Min Sun, Shandong Wu

**Affiliations:** 1 Department of Radiology, University of Pittsburgh School of Medicine, 4200 Fifth Ave, Pittsburgh, PA 15260 USA; 2 Department of Biomedical Informatics, University of Pittsburgh School of Medicine, 5607 Baum Blvd, Pittsburgh, PA 15206 USA; 3 Division of Oncology, University of Pittsburgh Medical Center Hillman Cancer Center at St. Margaret, 200 Delafield Rd, Pittsburgh, PA 15215 USA; 4 Department of Bioengineering, University of Pittsburgh, 4200 Fifth Ave, Pittsburgh, PA 15260 USA; 5 Intelligent Systems Program, University of Pittsburgh, 4200 Fifth Ave, 15260 PA Pittsburgh, USA

**Keywords:** Breast cancer, Radio-genomics, Machine learning, Tumor microenvironment, Cell type, Radiomics

## Abstract

**Background:**

The abundance of immune and stromal cells in the tumor microenvironment (TME) is informative of levels of inflammation, angiogenesis, and desmoplasia. Radiomics, an approach of extracting quantitative features from radiological imaging to characterize diseases, have been shown to predict molecular classification, cancer recurrence risk, and many other disease outcomes. However, the ability of radiomics methods to predict the abundance of various cell types in the TME remains unclear. In this study, we employed a radio-genomics approach and machine learning models to predict the infiltration of 10 cell types in breast cancer lesions utilizing radiomic features extracted from breast Dynamic Contrast Enhanced Magnetic Resonance Imaging.

**Methods:**

We performed a retrospective study utilizing 73 patients from two independent institutions with imaging and gene expression data provided by The Cancer Imaging Archive (TCIA) and The Cancer Genome Atlas (TCGA), respectively. A set of 199 radiomic features including shape-based, morphological, texture, and kinetic characteristics were extracted from the lesion volumes. To capture one-to-one relationships between radiomic features and cell type abundance, we performed linear regression on each radiomic feature/cell type abundance combination. Each regression model was tested for statistical significance. In addition, multivariate models were built for the cell type infiltration status (i.e. “high” vs “low”) prediction. A feature selection process via Recursive Feature Elimination was applied to the radiomic features on the training set. The classification models took the form of a binary logistic extreme gradient boosting framework. Two evaluation methods including leave-one-out cross validation and external independent test, were used for radiomic model learning and testing. The models’ performance was measured via area under the receiver operating characteristic curve (AUC).

**Results:**

Univariate relationships were identified between a set of radiomic features and the abundance of fibroblasts**.** Multivariate models yielded leave-one-out cross validation AUCs ranging from 0.5 to 0.83, and independent test AUCs ranging from 0.5 to 0.68 for the multiple cell type invasion predictions.

**Conclusions:**

On two independent breast cancer cohorts, breast MRI-derived radiomics are associated with the tumor’s microenvironment in terms of the abundance of several cell types. Further evaluation with larger cohorts is needed.

**Supplementary Information:**

The online version contains supplementary material available at 10.1186/s12885-021-08122-x.

## Background

Studies have shown that prognostic outcomes of tumors are not only linked with genetic factors within cancerous cells, but also with the extent of infiltrating immune and stromal cells in the tumor microenvironment (TME) [1]. For example, CD8 T cell infiltration in breast cancer has been associated with reduction in the relative risk of death from the cancer [2]. Additionally, in breast cancer, macrophage and endothelial cell presence are associated with reduced disease-free survival [3]. In clinical practice, biopsies and pathology are the most commonly used option to measure the presence of immune and stromal cells.

Radiomics is a field of study that aims to extract quantitative imaging features from radiological images for use in disease characterization. Radiomic features capture high-dimensional quantitative phenotypes in imaging data that are beyond what a radiologist can normally perceive via visual assessment. Radiomics were reported to predict molecular classification of breast malignancy, breast cancer recurrence risk, and many other disease outcomes [4–7]. Quantitative radiomics has shown the potential to be used as non-invasive imaging biomarkers to characterize tumor’s diagnosis, progression/prognosis, and treatment response [8–10]. Yet, the ability of radiomics methods to predict the abundance of various cell types in the TME remains unclear. Compared to the biopsy and pathology methods, the radiomics method is non-invasive, speedy, and repeatable to predict cell type invasion.

In this study, we employed a radio-genomics approach and machine learning models utilizing breast Dynamic Contrast Enhanced Magnetic Resonance Imaging (DCE-MRI) radiomic features to predict the presence of cell type invasion within breast cancer lesions. Radiomics can identify potential non-invasive predictors that characterize how immune and stromal cell infiltration may affect a tumor’s visible phenotype in radiological images. This would also lead to biological level of interpretability of radiomic features.

## Methods

### Study cohort

This retrospective study utilized imaging and gene expression data provided by The Cancer Imaging Archive (TCIA) [11] and The Cancer Genome Atlas (TCGA) [12], respectively. The imaging and clinical data were de-identified by TCIA and approved by the Institutional Review Board of the TCIA hosting institution. The study cohort was comprised of 73 patients from two independent institutions (43 and 30 patients from Institution 1 and Institution 2, respectively), and all the patients had both imaging data and gene expression information describing the lesion. The level 3 gene expression data for the 73 breast cancer lesions was downloaded from TCGA. The expression data took the form of the normalized counts of 20,530 genes obtained with Illumina Genome Analyzer Sequencing version 2.

Breast DCE-MRI data for the 73 patients were jointly downloaded from TCIA. All MRI sequences were acquired with a 1.5 Tesla magnetic field strength GE medical systems scanner. All the DCE-MRIs were comprised of one pre- and three post-contrast image series obtained using a T1-weighted three-dimensional spoiled gradient echo sequence and a gadolinium-based contrast agent. The average in-plane image resolution was 0.70 mm (range: 0.53 to 0.86). The 43 and 30 MRI sequences from Institution 1 and Institution 2 had a slice thickness of 2 mm and 2.2 mm with an image size of 512 × 512 pixels and 256 × 256 pixels, respectively.

**Fig. 1:**
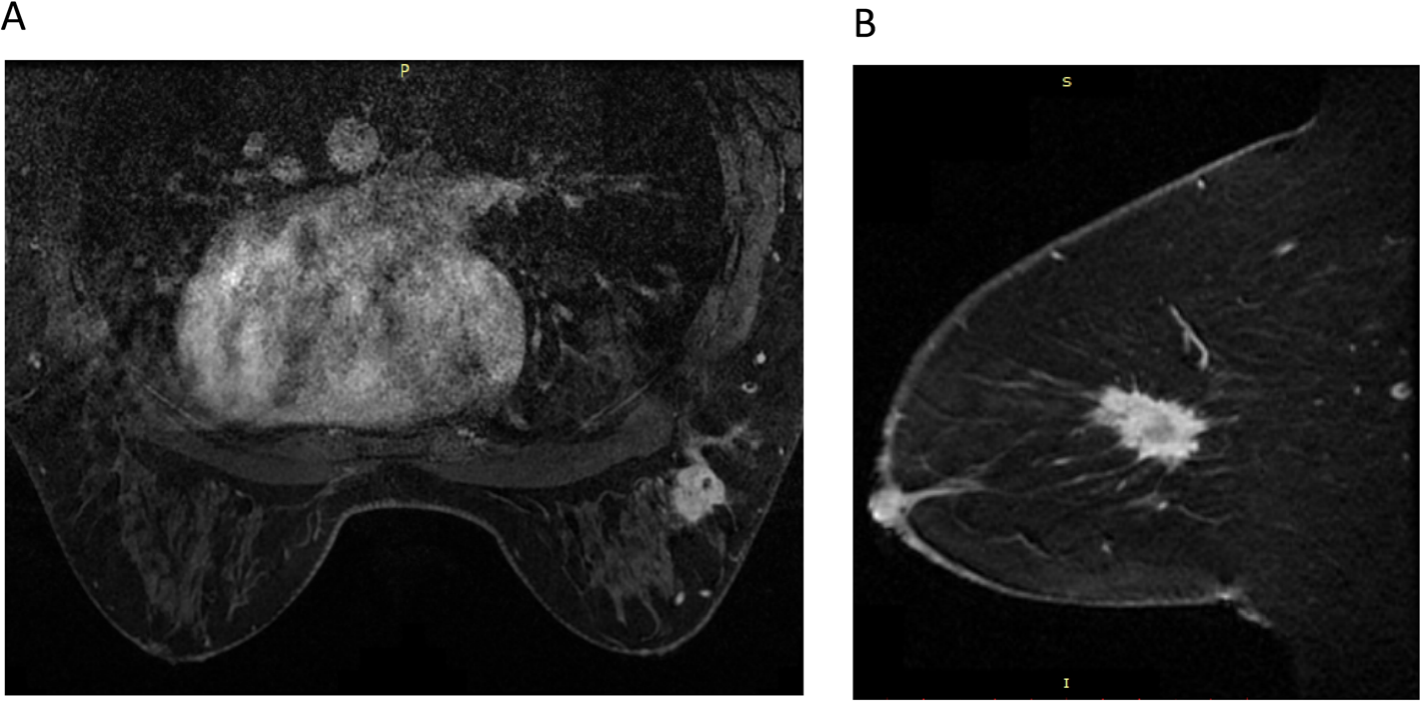
Representative images from Institution 1 (**a**) and Institution 2 (**b**) [independent evaluation set]

All sequences from Institution 1 used the axial view, while all sequences from Institution 2 used the sagittal view (Fig. 1). Due to differences in image collection protocol between institutions, we used Institution 1 data for radiomics modeling and Institution 2 data for external validation.

### Tumor segmentation and radiomic features

Prior to radiomic imaging feature extraction, segmentation of the tumor area is required for separation of the tumor region of interest from the normal breast tissue area in the MR images. Existing tumor segmentations performed by expert radiologists of these 73 cases were downloaded from The TCGA Breast Phenotype Research Group [13]. A set of 111 radiomic features were extracted from the lesion volumes using the PyRadiomics open-source Python package [14]. The radiomic features captured information related to tumor size (e.g. volume, surface area, maximum 3D diameter, and major axis length, etc.), morphological (e.g. elongation, flatness, sphericity, etc.), and texture (e.g., energy, entropy, kurtosis, skewness, gray level size zone matrix [GLSZM], gray level dependence matrix [GLDM], gray level Co-occurrence matrix [GLCM], and gray level run length matrix [GLRLM], etc.) properties. All images were rescaled to 8 bits/voxel and gray level quantization was performed using a fixed bin width (binWidth = 25). More details on the radiomic feature calculation in PyRadiomics including input parameters (e.g., distance and angles) were specified in Additional file 1.

In addition, the pre-contrast and all post-contrast sequences were used to calculate three categories of kinetic features (88 features in total): aggregate, heterogeneity, and textural kinetic features, using previously validated algorithms [15]. Aggregate kinetic features were calculated directly from the characteristic kinetic curve of each voxel over the tumor region. These features include: wash-in slope (WIS), wash-out slope (WOS), peak enhancement (PE), time to peak (TTP), maximum PE (MPE), and hot spot (the highest average of connected voxels). To calculate heterogeneity kinetic features, the segmented tumor was first partitioned into three sub-clusters according to their TT*P* values. The three sub-clusters (denoted as TTP characteristic maps) were formed by categorizing each pixel to represent quick, intermediate, and slow arrivals to the peak point [15]. The heterogeneity kinetic features were calculated on the TTP characteristic maps, using the mean and standard deviation (SD) of kinetic parameters (i.e. WIS, WOS, and PE) and the proportion of pixels in each of the three sub-clusters (e.g. mean quick WIS, mean intermediate WIS, and mean slow WIS, etc.). We also computed typical textural features on the above-created TTP characteristic maps (Textural kinetic features, e.g. Inverse Difference Moment [IDM], Absolute Value of Differences [AVD], contrast, skewness, Kurtosis, and Angular Second Moment [ASM], etc.)

### Cell type abundance quantification

Cell type abundance in the tumor was quantified using gene expression values and the R package Microenvironment Cell Populations-counter (MCP-counter) v 1.1.0 [16]. From a gene expression matrix, MCP-counter was able to produce an abundance score for eight immune cells (i.e. CD3+ T cells, CD8+ T cells, cytotoxic lymphocytes, NK cells, B lymphocytes, cells originating from monocytes, myeloid dendritic cells, neutrophils) and two stromal cells (i.e. endothelial cells, fibroblasts). DCE-MRI radiomic features were used to predict the abundance of these 10 cell types within breast cancer.

### Modeling and statistical analysis

To capture one-to-one relationships between radiomic features and cell type abundance, we performed linear regression on each radiomic feature/cell type abundance combination. Each regression model was tested for statistical significance. *P* values for each linear regression model were corrected for multiple comparisons using the Benjamini & Hochberg False Discovery Rate method [17]. To study any larger patterns in the relationships, we obtained the correlations between every radiomic feature/cell type abundance combination.

To build multivariate models for the cell type infiltration status prediction from breast MRI radiomic features, cell type abundance was stratified as binary classification, i.e., high or low, based on the median abundance of each cell type. Infiltration status for each cell type was stratified as “high” if the corresponding abundance score was equal or greater than the median abundance score of each cell type across the entire data set; otherwise it was stratified as “low”. A feature selection process was applied to the 199 radiomic features in order to pre-select a subset of discriminative features and to reduce data dimension for modeling. The feature selection was performed via Recursive Feature Elimination [18], implemented by the R package Caret v 6.0–81. The Recursive Feature Elimination model functions by iteratively removing features and testing the model’s performance using a random forest algorithm. The algorithm was tasked with finding the best performing set of features by maximizing the model’s accuracy on the training set. The radiomic classification models took the form of a binary logistic extreme gradient boosting framework. The models were implemented with the R package xgboost v 0.81.0.1 [19]. Two evaluation methods were used for radiomic model learning and testing: 1) using the 43 lesions from Institution 1 for training and testing under the typical leave-one-out cross validation strategy, and 2) training the models using 43 lesions from Institution 1 and testing the model independently using the 30 lesions from Institution 2. The models’ performance was measured via area under the receiver (AUC) operating characteristic curve.

We demonstrated the ability of two radiomic features to distinguish high and low cell type abundance. The two selected radiomic features are as follows: the volume of the tumor, a quantitative phenotype what is normally observed via visual assessment, and the mean peak enhancement of the tumor, the high-dimensional quantitative phenotype that is beyond what a radiologist can normally perceive via visual assessment. Ten two-sided t-tests were performed for the two radiomic features. Adjusted *p* values less than 0.05 were considered significant.

## Results

Study cohort statistics are displayed in Table 1. The average patient age at diagnosis was 54 ± 11.6 years. All patients were female, 40 (55%) of whom were pre-menopausal, 25 (34%) of whom were post-menopausal, and 8 (11%) of whom are unknown menopausal status. There are 18, 45, and 10 patients who had stage I, II, and III breast cancer, respectively. As we mentioned earlier, all sequences from Institution 1 were in the axial view (Fig. 1a) while all sequences from Institution 2 were in the sagittal view (Fig. 1b).

**Table 1:** Descriptive statistics describing study cohort

Total: *n* = 73			
Age (Years)			
Mean ± Standard Deviation	54 ± 11.6		
Molecular Receptor Statuses			
Receptor Status	Positive	Negative	Unknown
ER^a^	61	12	0
PR^b^	64	9	0
HER2^c^	8	41	24
Cancer Stages			
Stage I	18	–	
Stage II	45	–	
Stage III	10	–	
Menopausal Status			
Pre	40	–	
Post	25	–	
Unknown	8	–	

^a^ *ER* Estrogen receptors, ^b^ *PR* Progesterone receptors, ^c^ *HER2* Human epidermal growth factor receptor 2

Table 2 shows significant univariate linear associations between radiomic features and fibroblast cell type abundance. Only the fibroblast cell type showed any significant associations with radiomic features. Notably, every association was with a kinetic or texture-based feature.

**Table 2:** Statistically significant univariate associations between radiomic features and Fibroblast cell type abundance

Radiomic Feature	Feature Type	Correlation Coefficient	Adjusted p Value^**1**^
Tumor mean pixel intensity in Pre-contrast	Texture	0.42	0.011
Tumor mean pixel intensity in Post-contrast1	Texture/Kinetic	0.38	0.013
Tumor mean pixel intensity in Post-contrast2	Texture/Kinetic	0.39	0.011
Tumor mean pixel intensity in Post-contrast3	Texture/Kinetic	0.39	0.011
Mean intermediate WOS	Kinetic	0.39	0.011
Mean quick WIS	Kinetic	0.43	0.011
Mean intermediate WIS	Kinetic	0.41	0.011
Mean slow WIS	Kinetic	0.39	0.011
Mean quick PE	Kinetic	0.41	0.011
Mean intermediate PE	Kinetic	0.41	0.011
Mean slow PE	Kinetic	0.39	0.011
Tumor contrast	Texture	0.24	0.023
Tumor skewness in Post-contrast2	Texture/Kinetic	−0.38	0.013
Tumor kurtosis in Post-contrast3	Texture/Kinetic	−0.34	0.045

^1^ P values were adjusted for multiple comparisons using the Benjamini & Hochberg false discovery rate (FDR) method

**Fig. 2:**
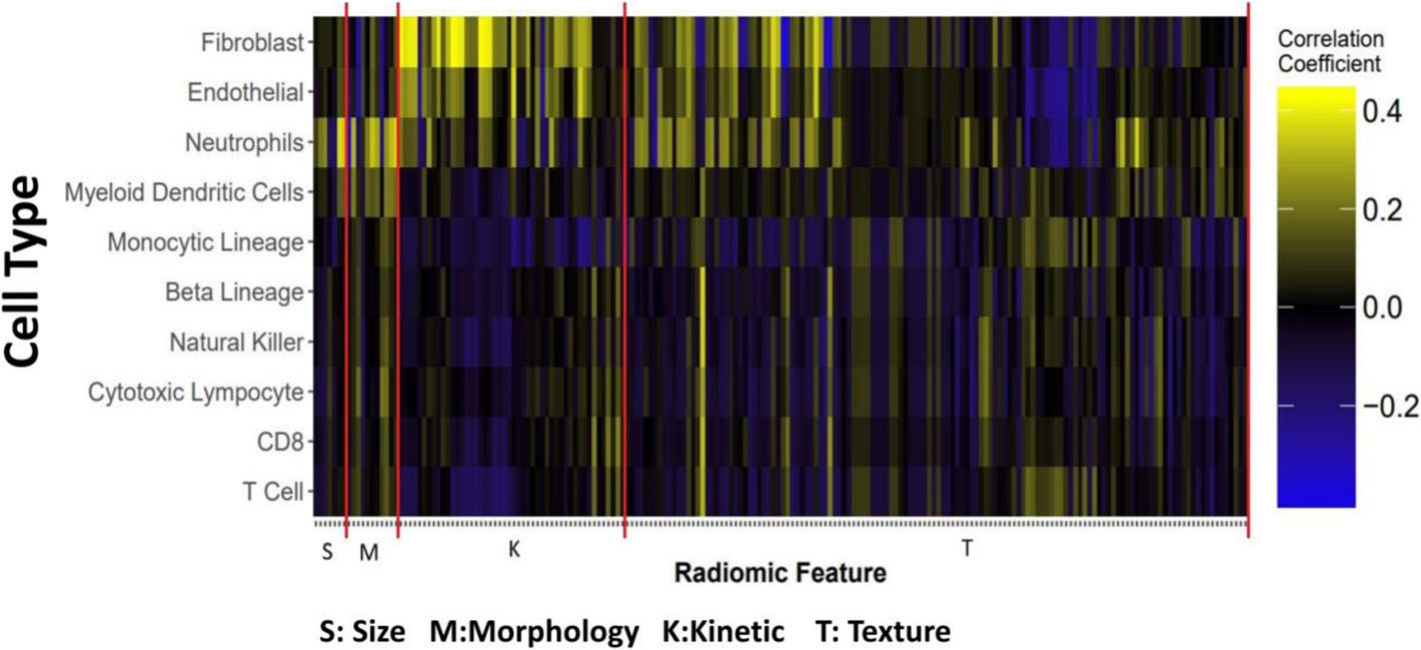
Relationships between immune and stromal cell abundance and radiomic features. Radiomic features are grouped into size, morphology, kinetic, and texture features

A heatmap describing the correlations between each individual radiomic feature and the ten cell types abundances are shown in Fig. 2. Radiomic features are grouped by the general phenotypic properties that they describe. It is observed that some radiomic features correlate uniformly across the cell type abundances, while most do not. As can be seen in Fig. 2, many of the size and morphology radiomic features, like diameter and perimeter, are positively correlated with neutrophil abundance. In contrast, fibroblasts and endothelial cells appear to mainly be correlated with kinetic features of the tumor. As can be seen in Table 2, the highest correlation is 0.43 between mean quick WIS and fibroblast cell type abundance. The highest negative correlation is − 0.38 between tumor skewness in the second Post-contrast MR sequence (Tumor skewness in Post-contrast2) and fibroblast cell type abundance.

**Fig. 3:**
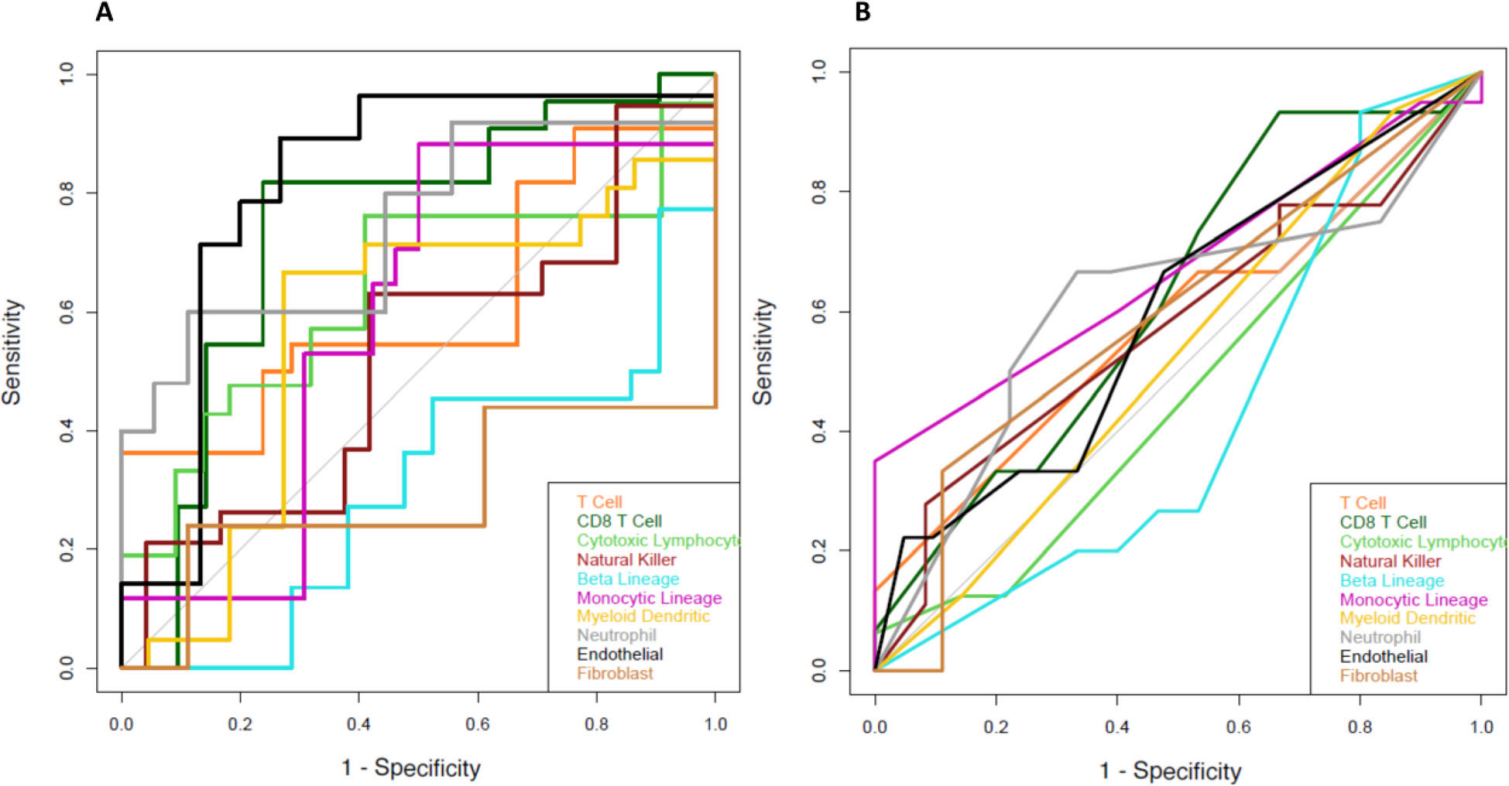
**a**) Leave-one-out cross validation (LOOCV) receiver operating characteristic (ROC) curves of 10 models predicting high/low cell type abundance for their corresponding 10 cell types on the data from Institution 1. **b**) ROC curves of the same 10 models predicting cell type abundance, tested on the independent data from Institution 2

**Fig. 4:**
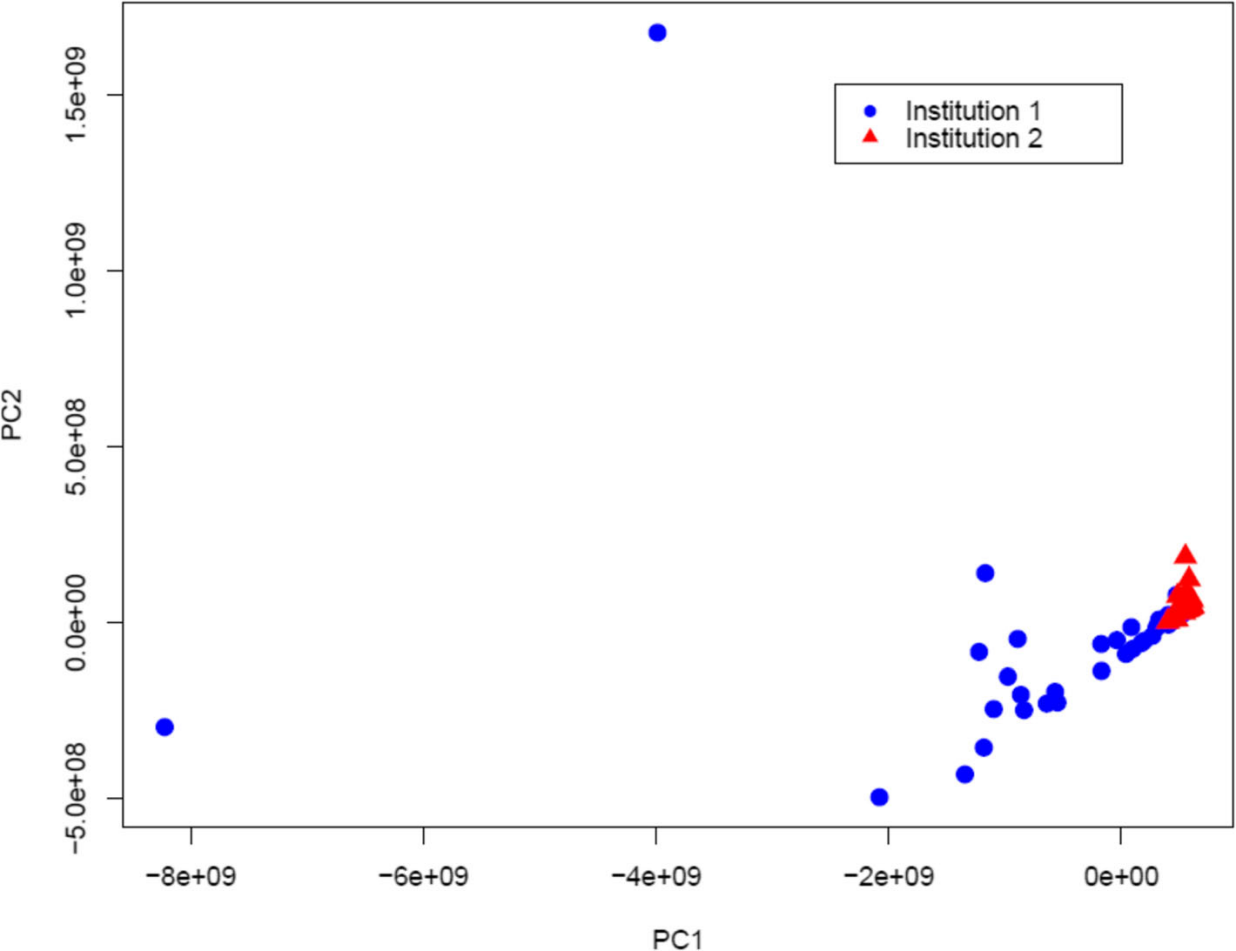
Principal component analysis to examine the separation of the radiomic features extracted from the two MRI datasets collected from different institutions

Feature selection chose a range of 3 to 16 radiomic features to include in the multivariate models. The most commonly selected features were the mean quick and intermediate PE, each appearing in four of the ten models. The full results on multivariate model’s AUC and receiver operating characteristic (ROC) curves are shown in Table 3 and Fig. 3, respectively. The models predicting high and low cell type abundance for ten cell types yielded leave-one-out cross validation AUCs ranging from 0.5 to 0.83. The same models tested on Institution 2 yielded AUCs ranging from 0.5 to 0.68. The average decrease from the leave-one-out AUC to the independent-test AUC is 0.185. Figure 4 shows the potential difference of radiomic features extracted from the two different MRI datasets, where the principal component analysis (PCA) indicates a separation between the two sets of radiomic features. Models with notably higher AUCs (i.e., AUC ≥ 0.80) include the models predicting NK cells and Neutrophils.

**Table 3:** Area under the ROC curve (AUC) values of the models

Cell type	LOOCV^a^ (43 patients from Institution 1)	Independent Test (30 patients from Institution 2)
T Cell	0.61	0.58
CD8 T Cell	0.74	0.62
Cytotoxic Lymphocyte	0.63	0.46
NK Cell	0.83	0.47
B Lineage	0.5	0.52
Monocytic Lineage	0.65	0.67
Myeloid Dendritic Cells	0.5	0.5
Neutrophils	0.82	0.61
Endothelial	0.77	0.68
Fibroblast	0.71	0.59

^a^ Leave one out cross validation

**Fig. 5:**
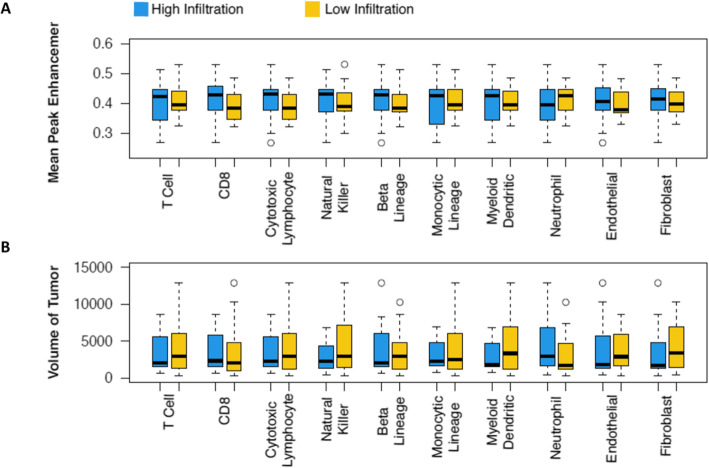
Differentiating high and low cell type infiltration with changes in **a**) Mean Peak Enhancement **b**) Volume of Lesion

Box and whisker plots showing how the two radiomic features differ between high and low cell type abundance are shown in Fig. 5. As can be seen, the volume of the lesion, a simple feature used by many radiologists, is unable to distinguish high and low cell type abundance for any cell types. There is, however, significant difference between high and low monocytic lineage (*p* = 0.004) and endothelial cell abundance (*p* = 0.05) when stratified by Mean PE, which is a more abstract radiomic feature describing how rapidly a contrast agent is absorbed into the lesion over time.

## Discussion

In this study, our analyses show that breast DCE-MRI-derived radiomics (from macroscopic imaging) are associated with invasion of several immune and stromal cells in breast cancer lesions. This study contributes to understanding the relationships between a breast tumor microenvironment and its radiological phenotypes. Although the analyses are preliminary due to the constraints of sample size, the findings, if validated on larger cohorts, have important clinical implications. It indicates that quantitative radiomics features extracted from standard-of-care radiological images may provide a non-invasive, cost-effective, and speedy way to characterize breast tumor’s microenvironment (i.e. abundance of different cell types). This may contribute to augmenting the clinical prognostication of breast cancer patients.

The correlations between cell type abundance and radiomic features shown in Fig. 2 suggest that in general, radiomic features describing size are not as informative of cell type abundance in tumors as morphological, kinetic, or texture-based radiomic features. These are the features that are not effectively or possibly assessed by human visual observations. The boxplots in Fig. 5 further support this point by showing how mean PE, a kinetic feature, can differentiate high and low abundance of two cell types, while volume of tumor displays no differences.

As shown in Table 2, Fibroblasts showed encouraging significant univariate relationships. A possible explanation for Fibroblasts showing the strongest associations may be due to that Fibroblast cells are critical in the development of connective tissue [20]. Following this line of thinking, connective tissue might be a more visible aspect of the tumor as compared to individual cells. While many correlations do appear to exist between cell type abundance and radiomic features, the number of statistically significant univariate relationships are fewer. This could likely be due to our relatively small sample size that does not have adequate statistical power.

The multivariate models predicting endothelial cell, CD8+ T cell, and neutrophils are the most promising (Table 3). In a recently published article [21], a multivariate model was built to predict the abundance of CD8+ T cell using radiomic features extracted from tumor in contrast-enhanced CT images. The reported AUC value was 0.67, which is in line with our multivariate analysis for CD8+ T cell abundance prediction (AUC = 0.74 and 0.62 for leave-one-out cross validation and external independent test, respectively). These findings indicate the robustness of radiomic features, regardless extracted from MRI or CT images, in reflecting the association with the cell type of CD8 + .

While moderate loss in the AUC between leave-one-out testing and independent testing is observed, these can be interpreted as expected results. One of the potential reasons can relate to the fact that the MRI images used for the training and testing sets come from two separate institutions and undergo different image acquisition protocols. In addition to the difference that all images acquired from Institution 1 are in axial view and all images from Institution 2 are in sagittal view (Fig. 1), the difference in image resolution, MRI contrast agent, and other imaging parameters can lead to large impact on radiomic feature calculation and thus model’s performance. The PCA analysis results shown in Fig. 4 have indicated the separation of the two sets of radiomic features extracted from the two institutions’ MRIs.

Our study has a few limitations and points to important follow-up studies. Firstly, since gene expression data are not routinely available for most of the breast cancer patients, our study was limited to a relatively small number of patients who had both imaging and gene expression data available for such analysis; yet, we were able to include two independent datasets from different institutions. Also, while we have used the MCP-counter method to quantify the abundance of the cell populations, it would be better to use the more robust immunohistochemistry (IHC) staining techniques on tumor sections to further validate our findings. The IHC data of the study cohorts were not available to perform such analyses. Secondly, the radiomics features used in our study may not be comprehensive but we intended to keep the feature set compact, considering our sample size of study cohort. In addition, more sophisticated methods to reduce inconsistency of the imaging characteristics across data from different institutions are in need and a goal of our future research. Finally, because of the preliminary nature of this study, we have been cautious in interpreting the biological rationales of the identified relationships, but our findings show evidences that the quantitative phenotypes in radiological images may potentially serve as surrogate markers of the cells’ abundance to inform patient prognostication. Paired with prognostic outcomes, like recurrence or response to immunotherapy, it merits further investigation on how radiomics and pathology may augment each other in functionally characterizing lesions, and ultimately lead to improved patient care using the validated imaging phenotype biomarkers.

## Conclusions

In this study of using two small but independent breast cancer cohorts, we show that breast MRI-derived radiomics are associated with the tumor’s microenvironment in terms of the abundance of several cell types. While the reported findings warrant further evaluation on larger cohorts, this study points to the potential value of quantitative radiomics as a non-invasive imaging biomarker to augment clinical prognostication of breast cancer patients.

## Supplementary Information

**Additional file 1.** The list of higher order statistical features and input parameters.

## Data Availability

The data are publicly available through TCIA website: https://www.cancerimagingarchive.net/access-data/

## Abbreviations

TME: Tumor microenvironment
DCE-MRI: Dynamic Contrast Enhanced Magnetic Resonance Imaging
TCIA: The Cancer Imaging Archive
TCGA: The Cancer Genome Atlas
AUC: Area under the receiver
GLSZM: Gray level size zone matrix
GLDM: Gray level dependence matrix
GLCM: Gray level co-occurrence matrix
GLRLM: Gray level run length matrix
WIS: Wash-in slope
WOS: Wash-out slope
PE: Peak enhancement
TTP: Time to peak
MPE: Maximum PE
SD: Standard deviation
IDM: Inverse Difference Moment
AVD: Absolute Value of Differences
ASM: Angular Second Moment
MCP: Microenvironment Cell Populations
