# Supplementary material for: Predicting cell invasion in breast tumor microenvironment from radiological imaging phenotypes
Source: BMC Cancer. 2021 Apr 7;21:370. doi: 10.1186/s12885-021-08122-x (PMC8028733; doi:10.1186/s12885-021-08122-x)
Supplement: Supplementary file 1 — Additional file 1. The list of higher order statistical features and input parameters. [file 12885_2021_8122_MOESM1_ESM.docx]

**Additional file 1**

Mathematical definitions of the radiomic features extracted using PyRadiomics are given on the PyRadiomics documentation page (<https://pyradiomics.readthedocs.io/en/latest/features.html>).

The list of higher order statistical features and input parameters used for computations are as follows:

- **Gray Level Co-occurrence Matrix (GLCM) Features: (24 features)**

GLCM features were calculated for each angle separately and the mean of these values over all angles was returned.

Specific settings:

- The distance from the center voxel= 1 pixel
- 13 and 4 angles (26 and 8-connectivity) in 3D and 2D were used, respectively. (e.g. θ = 0°, 45°, 90°, and 135° for 2D)
- Co-occurrences were assessed in two directions per angle. (symmetricalGLCM: True)

1. **Autocorrelation**
2. **Joint Average**
3. **Cluster Prominence**
4. **Cluster Shade**
5. **Cluster Tendency**
6. **Contrast**
7. **Correlation**
8. **Difference Average**
9. **Difference Entropy**
10. **Difference Variance**
11. **Joint Energy**
12. **Joint Entropy**
13. **Informational Measure of Correlation (IMC) 1**
14. **Informational Measure of Correlation (IMC) 2**
15. **Inverse Difference Moment (IDM)**
16. **Maximal Correlation Coefficient (MCC)**
17. **Inverse Difference Moment Normalized (IDMN)**
18. **Inverse Difference (ID)**
19. **Inverse Difference Normalized (IDN)**
20. **Inverse Variance**
21. **Maximum Probability**
22. **Sum Average**
23. **Sum Entropy**
24. **Sum of Squares**

- **Gray Level Run Length Matrix (GLRLM) Features: (16 features)**

GLRLM features were calculated for each angle separately and the mean of these values over all angles was returned.

Specific settings:

- 13 and 4 angles (26 and 8-connectivity) in 3D and 2D were used, respectively. (e.g. θ = 0°, 45°, 90°, and 135° for 2D)

1. **Short Run Emphasis (SRE)**
2. **Long Run Emphasis (LRE)**
3. **Gray Level Non-Uniformity (GLN)**
4. **Gray Level Non-Uniformity Normalized (GLNN)**
5. **Run Length Non-Uniformity (RLN)**
6. **Run Length Non-Uniformity Normalized (RLNN)**
7. **Run Percentage (RP)**
8. **Gray Level Variance (GLV)**
9. **Run Variance (RV)**
10. **Run Entropy (RE)**
11. **Low Gray Level Run Emphasis (LGLRE)**
12. **High Gray Level Run Emphasis (HGLRE)**
13. **Short Run Low Gray Level Emphasis (SRLGLE)**
14. **Short Run High Gray Level Emphasis (SRHGLE)**
15. **Long Run Low Gray Level Emphasis (LRLGLE)**
16. **Long Run High Gray Level Emphasis (LRHGLE)**

- **Gray Level Size Zone Matrix (GLSZM) Features: (16 features)**

Specific settings: N/A

1. **Small Area Emphasis (SAE)**
2. **Large Area Emphasis (LAE)**
3. **Gray Level Non-Uniformity (GLN)**
4. **Gray Level Non-Uniformity Normalized (GLNN)**
5. **Size-Zone Non-Uniformity (SZN)**
6. **Size-Zone Non-Uniformity Normalized (SZNN)**
7. **Zone Percentage (ZP)**
8. **Gray Level Variance (GLV)**
9. **Zone Variance (ZV)**
10. **Zone Entropy (ZE)**
11. **Low Gray Level Zone Emphasis (LGLZE)**
12. **High Gray Level Zone Emphasis (HGLZE)**
13. **Small Area Low Gray Level Emphasis (SALGLE)**
14. **Small Area High Gray Level Emphasis (SAHGLE)**
15. **Large Area Low Gray Level Emphasis (LALGLE)**
16. **Large Area High Gray Level Emphasis (LAHGLE)**

- **Neighboring Gray Tone Difference Matrix (NGTDM) Features: (5 features)**

NGTDM features are rotation independent.

Specific settings:

- The distance from the center voxel= 1 pixel

Contrary to GLCM and GLRLM, the GLSZM is rotation independent, with only one matrix calculated for all directions in the ROI.

1. Coarseness
2. Contrast
3. Busyness
4. Complexity
5. Strength

- **Gray Level Dependence Matrix (GLDM) Features: (14 features)**

Specific settings:

- The distance from the center voxel= 1 pixel
- Cutoff value for dependence: 0

1. **Small Dependence Emphasis (SDE)**
2. **Large Dependence Emphasis (LDE)**
3. **Gray Level Non-Uniformity (GLN)**
4. **Dependence Non-Uniformity (DN)**
5. **Dependence Non-Uniformity Normalized (DNN)**
6. **Gray Level Variance (GLV)**
7. **Dependence Variance (DV)**
8. **Dependence Entropy (DE)**
9. **Low Gray Level Emphasis (LGLE)**
10. **High Gray Level Emphasis (HGLE)**
11. **Small Dependence Low Gray Level Emphasis (SDLGLE)**
12. **Small Dependence High Gray Level Emphasis (SDHGLE)**
13. **Large Dependence Low Gray Level Emphasis (LDLGLE)**
14. **Large Dependence High Gray Level Emphasis (LDHGLE)**
